# Education Research: Trends in the National Resident Matching Program Headache Medicine Match Data: A Retrospective Observational Study

**DOI:** 10.1212/NE9.0000000000200202

**Published:** 2025-03-05

**Authors:** Niushen Zhang, Noah Rosen

**Affiliations:** 1 Department of Neurology, Stanford University School of Medicine, CA; and; 2 Zucker School of Medicine at Northwell Health, Hempstead, NY.

## Abstract

**Background and Objectives:**

The objective of this study was to examine the trends in the National Resident Matching Program (NRMP) Headache Medicine match data since inception and the evolution of the Headache Medicine fellowship application process. The NRMP Headache Medicine fellowship match was implemented for appointment years 2016–2018 and then suspended, in part because of the small number of applicants relative to available positions and participation outside of the match. In 2019, the American Headache Society (AHS) Consortium of Academic Program Directors accepted the American Academy of Neurology (AAN) Fellowship Application Timing Position Statement. The Headache Medicine match was reinstated for appointment year 2023 and has continued since that time.

**Methods:**

We obtained NRMP match data for appointment years 2016, 2017, 2018, 2023, 2024, and 2025 with permission and assistance from the NRMP Data and Research department. Quantitative data were summarized with descriptive statistics. The study was reviewed and approved by Stanford University's Institutional Review Board (Protocol ID: 71480).

**Results:**

We compared the averages (for number of certified programs, certified positions, program fill rate, and certified applicants) of appointment years 2016–2018 with the averages of appointment years 2023–2025. There were 2.4 times more certified programs participating in the most recent 3 cycles of the match. There were 2.6 times more certified positions in the most recent 3 cycles of the match. The range of program fill rate was 26.7% (2017) to 81.0% (2025). The program fill rate nearly doubled (1.8 times greater) in the most recent 3 cycles of the match. For the number of certified applicants, the range was 7 (in 2017) to 61 (in 2025). There were 4.5 times more certified applicants in the most recent 3 cycles of the match.

**Discussion:**

The Headache Medicine fellowship application process has undergone a significant transformation from 2015 to 2024. The number of programs and applicants participating in the NRMP match increased significantly over time in the setting of implementing the AAN's unified application time line and launching the AHS National Headache Fellowship Opportunities website, and with increasing total number of Neurology and Child Neurology graduates.

## Introduction

The United Council for Neurological Subspecialties (UCNS) is an organization founded in 2003 to promote high-quality patient-centered neurologic care by the accreditation of training programs and certification of physicians in neurologic subspecialties.^1^ In 2005, Headache Medicine, along with Neuroimaging, Neuro-oncology, Neurocritical Care, and Clinical Neuromuscular Pathology, became approved for subspecialty recognition. The first programs in Headache Medicine were accredited in 2006, and the first certification examinations occurred that year. By 2016, annual reporting was launched for accredited programs and the first recertification examinations were offered. By 2020, recertification transitioned to a continuous certification process and the number of certified headache specialists has continued to grow.

There is high variability between the individual matching processes for each neurologic subspecialty. Several Accreditation Council for Graduate Medical Education (ACGME) and UCNS-recognized subspecialties have organized match processes through the National Resident Matching Program (NRMP): Brain Injury Medicine, Clinical Neurophysiology, Epilepsy, Pain Medicine, Sleep Medicine, Vascular Neurology, and now Headache Medicine. Some subspecialties, such as Neurocritical Care, Neuro-Oncology, and Movement Disorders, use the San Francisco Match. Starting in 2021, Neuromuscular Medicine developed its own internal application and match process unique from any other specialty. Other fellowship pathways such as Cognitive and Behavioral Neurology and Neuroimmunology still rely on more open-ended individual program directed agreements without external oversight.^2,3^

Given the perceived need within Headache Medicine after nearly 10 years of accredited programs, the Consortium of Academic Headache Program Director (sponsored by the American Headache Society [AHS]) agreed to initiate a certified match under the NRMP. Despite the high variability in what was performed in the UCNS vs ACGME-recognized specialties, this was determined to be the path most compatible with Headache Medicine. The NRMP Headache Medicine fellowship match was implemented for appointment years 2016–2018 and then was briefly suspended because of the small number of applicants relative to available positions and participation outside of the match. In 2019, the AHS Consortium of Academic Program Directors accepted the American Academy of Neurology (AAN) Fellowship Application Timing Position Statement.^4^ To implement the unified time line and to create a more centralized process, the AHS agreed to host a fellowship opportunities website for programs and applicants.^5^ Programs had to agree to participate in the unified time line to be featured on the website. This website provides direct, updated communication to programs and participants, ensuring that applicants are aware of whom to contact and to ensure an easy flow for the application process. If a program was not participating in the match, or had actions that violated the agreement, it would be removed from the website for that match year.^6^

In calendar years 2020 and 2021, the Consortium sponsored an internal match process. This agreement between programs instituted a spring interview season followed by an August match day when programs could reach out to their primary choices. This workflow was well received but demonstrated specific flaws that suggested that this process was not optimal. There was no standardization of the process, risk of undue pressures on the candidate to make a decision immediately, and a nonoptimized process matching candidates to positions. In 2022, the Consortium voted to reinstate the NRMP match, which has continued since. The purpose of this study was to examine the trends in the NRMP Headache Medicine match data and the evolution of the Headache Medicine fellowship application process.

## Methods

### Data Collection

We obtained NRMP match data for appointment years 2016, 2017, 2018, 2023, 2024, and 2025 with permission and assistance from the NRMP Data and Research department. The Stanford University Institutional Review Board reviewed and approved the protocol (Protocol ID: 71480).

### Statistical Analysis

Data were extracted into a Microsoft Excel file. The data were summarized with descriptive statistics, such as frequency and percentages.

### Data Availability

The data that support the findings of this project are available from the corresponding author on reasonable request.

## Results

**Figure 1:**
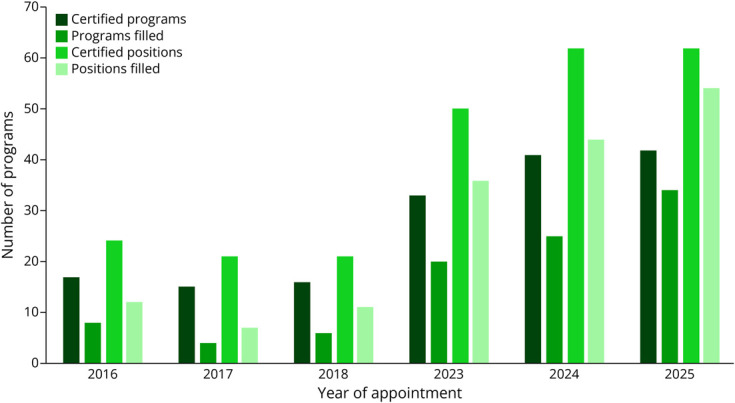
Program Data From the National Resident Matching Program Headache Medicine Match

**Figure 2:**
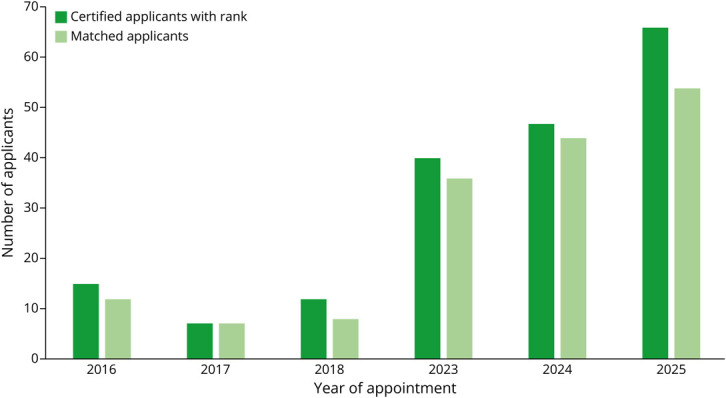
Applicant Data From the National Resident Matching Program Headache Medicine Match

**Figure 3:**
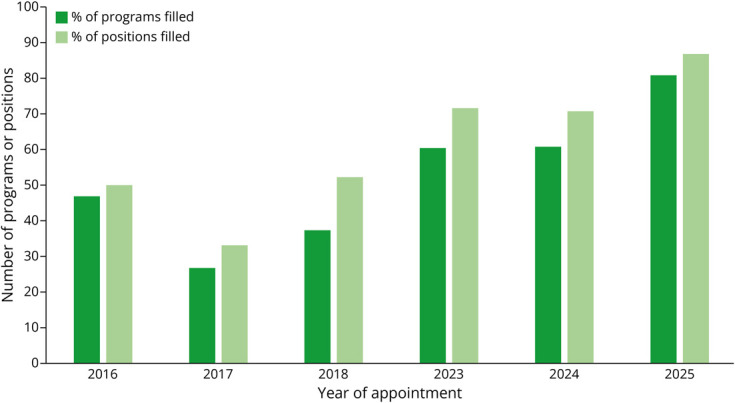
Program and Position Fill Rate

**Figure 4:**
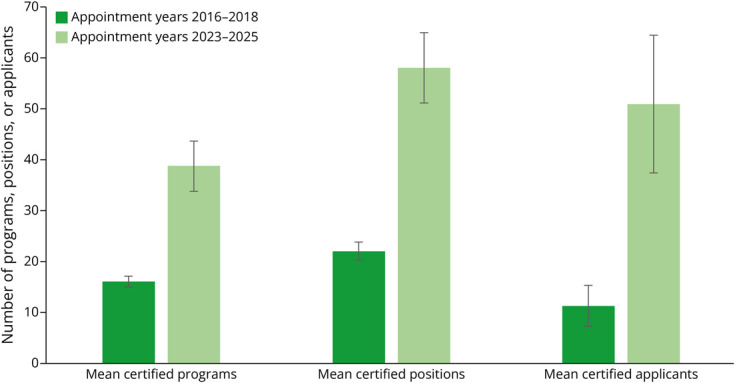
Comparison of Data for Appointment Years 2016–2018 to 2023–2025

Using descriptive statistics, we analyzed the NRMP match data for appointment years 2016, 2017, 2018, 2023, 2024, and 2025 (Table 1, Figures 1–3). Given that there was a 4-year break between the first 3 cycles and the second 3 cycles, we compared the averages (for number of certified programs, certified positions, program fill rate, and certified applicants) of appointment years 2016–2018 with the averages of appointment years 2023–2025 (Table 2, Figure 4).

**Table 1:** National Resident Matching Program Headache Medicine Match Results

Program statistics	Year of appointment					
	2016	2017	2018	2023	2024	2025
Certified programs	17	15	16	33	41	42
Programs filled	8 (47.1%)	4 (26.7%)	6 (37.5%)	20 (60.6%)	25 (61.0%)	34 (81.0%)
Programs unfilled	9 (52.9%)	11 (73.3%)	10 (62.5%)	13 (39.4%)	16 (39.0%)	8 (19.0%)
Certified positions	24	21	21	50	62	62
Positions filled	12 (50.0%)	7 (33.3%)	11 (52.4%)	36 (72.0%)	44 (71.0%)	54 (87.1%)
Positions unfilled	12 (50.0%)	14 (66.7%)	10 (47.6%)	14 (28.0%)	18 (29.0%)	8 (12.9%)
Applicants per position	0.625	0.333	0.571	0.80	0.758	1.065
Applicant statistics						
Certified applicants with rank	15	7	12	40	47	66
Total certified US MD graduates	8 (53.3%)	5 (71.4%)	8 (66.7%)	23 (57.5%)	27 (57.4%)	36 (54.5%)
Total certified DO graduates	NA	NA	NA	10	8	15 (22.7%)
Total certified US international graduates	NA	NA	NA	3	3	4 (6.1%)
Total certified non-US international graduates	NA	NA	NA	4	7	11 (16.7%)
Matched applicants	12 (80.0%)	7 (100.0%)	11 (91.7%)	36 (90.0%)	44 (93.6%)	54 (81.8%)
US MD graduates	6	5	8	23	26	31
DO graduates	4	0	0	10	8	14
US international graduates	0	2	2	1	3	3
Non-US international graduates	2	0	1	2	6	6

**Table 2:** Comparison of Match Data From Appointment Years 2016–2018 and 2023–2025

Appointment years	Range	Median	Mean	SD
2016–2018				
Certified programs	15–17	16	16	1.0
Certified positions	21–24	21	22	1.7
Certified applicants	7–15	12	11.3	4.0
Program fill rate, %	26.7–47.1	37.5	37.1	10.2
2023–2025				
Certified programs	33–42	41	38.7	4.9
Certified positions	50–62	62	58	6.9
Certified applicants	40–66	47	51	13.5
Program fill rate, %	60.6–81.0	61.0	67.1	11.7

For the number of certified programs participating in the match from appointment years 2016 to 2025, the range was 15 (in 2017) to 42 (in 2025). The average number of certified programs across periods (2016–2018 vs 2023–2025) increased by 2.4-fold (16 vs 38.7). The range of certified positions was 21 (in 2017 and 2018) to 62 (in 2025). The average number of certified positions across the periods increased by 2.6-fold (58 vs 22). The range of program fill rate was 26.7% (2017) to 81.0% (2025). The average program fill rate across periods nearly doubled (37.1% vs 67.1%). Regarding the number of certified applicants, the range was 7 (in 2017) to 61 (in 2025). The average number of certified applicants across the periods increased by 4.5-fold (11.3 vs 51).

In addition, the applicant match rate was greater than 90% in all years except for appointment year 2016. The 2 most recent cycles of the match saw a significant rise in the number of matched DO applicants, from none in appointment years 2017 and 2018 to 10 in appointment year 2023 and 14 in appointment year 2025. Regarding notable findings for the most recent match cycle (appointment year 2025), the number of positions remained the same as appointment year 2024 but the number of certified programs increased by 1. The number of certified applicants increased to an all-time high of 66 (from 47 for appointment year 2024). The program fill rate increased to an all-time high of 81.0% (from 61.0% for appointment year 2024). There were half as many unfilled programs as for appointment year 2024. This was the first time in the history of the Headache Medicine match where there is more than 1 applicant for each certified position (1.065 applicants per position).

## Discussion

Headache Medicine is a rapidly evolving field. Since the beginning of accreditation and certification under the UCNS in 2006, it has continued to attract more practitioners from a range of different specialties. As of 2024, there are 54 accredited Headache Medicine fellowship programs in the United States. As of the end of 2023, there are 729 certified specialists, a number that has only continued to grow since the program's inception. While the largest number of those certified specialists became eligible for the examination through the clinical pathway (recognizing work that has been conducted in the area for years), fellowship-trained experts are rapidly increasing. While no specific plans have been made to close the clinical pathway, it is likely that fellowship training will become the primary pathway within the next few years.

Given the increasing number of programs since the start of UCNS accreditation and the consequent increase in the number of training positions available, the process for ensuring those positions were filled became paramount. This was brought up in multiple discussions at the Academic Headache Program Director Consortium at the AHS (which became the Headache Fellowship Director Committee of the AHS in 2022). Comparisons were made with other Neurology fellowships, and a wide array of processes was demonstrated. Currently, the ACGME/Neurology-recognized subspecialties include the following: Brain Injury Medicine, Clinical Neurophysiology, Epilepsy, Neurocritical Care, Neurodevelopmental Disabilities, Neuroendovascular Intervention, Neuromuscular Medicine, Pain Medicine, Sleep Medicine, and Vascular Neurology. The UCNS-recognized neurology subspecialties include Autonomic Disorders, Behavioral Neurology and Neuropsychiatry, Clinical Neuromuscular Pathology, Geriatric Neurology, Headache Medicine, Interventional Neurology, Neonatal Neurocritical Care, Neurocritical Care (the only specialty with 2 potential pathways), Neuroimaging, and Neuro-oncology. Several notable specialties have no external oversight for their training programs including Movement Disorders, Autoimmune Disorders, and others.

The increase in the number of certified programs, positions, and applicants and the program match rate all demonstrate significant growth in the field. However, despite development in this area, there are still not enough specialists to meet the needs of the population of patients with headache.^7^ Meeting the need may require rethinking the appropriate use of a headache-trained specialist and how this specialized group can be leveraged effectively. This current process has been predominantly about establishing a new field of practice and not simply perpetuating historical training programs. Over the past several decades, the field started with a small number of training programs, established a certification process, and eventually standardized the process to make it more clearly presented and user friendly. When the initial 3 cycles of the headache match occurred, there was limited success because of the small pool of applicants and the low participation in the match among programs. By the second set of 3 match cycles, the numbers show a different story. There has not just been a strong increase in the growth of programs and available positions but also a significant growth in the number of applicants. Over subsequent years, applications have continued to be strong and match rates continue to approach 100%.

A number of factors likely contributed to the significant increase that we saw in all areas of the match over the past 3 cycles. The field itself has seen significant changes including the introduction of new headache treatments (i.e., calcitonin gene–related peptide targeting treatments), which may have increased recognition and socialization and helped drive that change. The coronavirus disease 2019 pandemic created changes in lifestyle expectations, including the introduction of remote work, through telemedicine, which is very feasible with headache medicine. Certification itself helps define differentiation among applicants, and that value is likely to continue to rise. It is possible that we have reached a critical mass of headache specialists, including new graduates from fellowship programs who have started their own academic headache programs and self-propagated the field. Headache specialists have continually grown in academic and leadership positions not only in the professional societies such as AAN but also in local hospital systems.^8^ When the AHS Consortium of Academic Program Directors first met, most of the fellowship directors were also the heads of their respective headache centers. Now, those positions are often separated, with leadership positions occupied by different, more expansive, and diverse people.

In addition, the AHS launched a number of headache education initiatives in the past 10 years focused on expanding knowledge and interest in Neurology and Child Neurology residents and residents from other fields.^9^ Started in 2018, the annual AHS Resident Education Program brings prospective residents from all across the country together for a weekend of intensive headache education.^10^ The REACH program was also started in 2018 and has brought headache medicine–focused grand rounds and additional education to more than 70 institutions across the United States.^11^ This had been in addition to the longer existent International Headache Academy, which has annually brought together research-focused residents, fellows, and junior attendings.

There has been a culture change among the academic headache programs from the initial 3 cycles of the match to the most recent 3 cycles of the match. The shift in culture began in 2019, when the AHS Consortium of Academic Program Directors accepted the AAN Fellowship Application Timing Position Statement. At that point, more programs bought into the utilization of the unified application time line, which proved to be an important step in the field's transition back to the match. The AHS Fellowship Opportunities website also served as an important incentive that encouraged programs to participate in the unified application time line and the match to reach a greater audience of applicants. The website serves as a centralized resource for programs and applicants, creating a virtual community center that allows programs and applicants to be a part of a greater whole. The match creates a uniform application process and has been central to establishing a standardized process and a time line for maximizing access and increasing the total number of fellowship-trained headache specialists. The match process helps solidify and legitimize the field as a training option for long-term employment and to provide trainees with identity and individuation.

Looking to the future, the question will be, “How do we continue these trends from the last 3 cycles of the match?” The rate of growth of new Headache Medicine fellowship programs and available training positions has experienced a dramatic increase over the period reviewed in this article. As of December 2024, there are 58 programs on the UCNS website.^12^ Given that there are only 155 Neurology training programs in the country, it is reasonable to surmise that the rate of development of new headache training programs will reach a pinnacle and will likely stabilize at that point. However, given current headache and migraine prevalence, it is unlikely that enough headache specialists would be available if the field focuses on training only neurologists. To meet that gap, it is possible that recruitment may require looking beyond Neurology for additional providers. Already there are family medicine practitioners, internists, physical medicine and rehab specialists, gynecologists, and other specialists who practice in the field, and many are UCNS certified. Further opportunities for expansion would help ease the practice gap. There is no barrier to recruitment from these fields regarding UCNS certification because no specific primary training track is required for subspecialty certification.^13^ Another potential option for headache training programs may be a reinvisioning of the role of the headache specialist. Very much the way that other professions have modified their scope of practice, the role of a headache specialist may need to be modified to meet societal needs. Rather than direct patient care, more emphasis may be needed in supervising procedural technicians, ensuring that quality metrics are made, and increasing translational research or educational expectations. Training programs are likely to be the cauldron in which these initiatives are created. It will also be important to survey feedback from applicants and programs about their experience with the current process. We need to understand what parts of this process were most useful in attracting more applicants, what limitations there are, and will there be more opportunities and more programs in the future. We may also consider a centralized application process or change in time line if needed. In the future, further insights may be gleaned by incorporating UCNS data into the AHS website data to help us better understand the landscape.

This retrospective observational study, although valuable in identifying trends in the Headache Medicine fellowship application process, has limitations that should be noted. The study examines only a few match cycles (2016–2018, 2023–2025), potentially overlooking annual variations and broader historical trends that might provide additional context to the findings. In addition, there are limited data on applicants who did not match. The information collected and shared by the NRMP during the Headache Medicine application process is limited and does not include complete demographic data. Because NRMP only provides oversight for the match itself, individual data are separated from the actual application packet for each candidate, which is provided directly to each program. As such, that information is not collected and analyzed. This piecemeal process does not allow for a comprehensive “nose to tail” experience. By contrast, the Neuromuscular Medicine application uses a single proprietary integrated system, which is able to capture all of the information provided by candidates and allows for greater insights into the application process.^14^ In addition, it is also difficult to account for external influences, such as changes in health care policies or economic factors, which may also affect fellowship program interest and applicant numbers. Last, the study's focus on quantitative data restricts an in-depth understanding of applicants' motivations and program directors' perspectives, both of which could offer a more comprehensive view of factors driving growth and challenges within Headache Medicine.

The Headache Medicine fellowship application process has undergone a significant transformation from 2015 to 2024. It is now exemplary among smaller neurologic fellowships as a highly organized and systematic process with participation from most of the academic headache programs. The fellowship match process in Headache Medicine has continued to grow regarding candidates, programs, and available positions. This was in part accomplished by coordinated efforts between the AAN and the AHS in harmonizing the fellowship time line and focusing on a reasonable, understandable time line. While creating a standardized time line across fellowships was an AAN initiative, its implementation requires significant effort on the part of the subspecialty organization. The Headache Fellowship Director Committee was formed under the AHS and found a home at its regular meetings. This allowed for a centralized gathering place for discussion and accomplishing individual objectives that led to the goal of a formalized match process. The synergy between these organizations allowed the field of Headache Medicine to advance, and this may be applicable to multiple other subspecialties under the Neurology bailiwick.

## Glossary

AAN: American Academy of Neurology
ACGME: Accreditation Council for Graduate Medical Education
AHS: American Headache Society
NRMP: National Resident Matching Program
UCNS: United Council for Neurological Subspecialties
